# Development of a neuroprotective peptide that preserves survival pathways by preventing Kidins220/ARMS calpain processing induced by excitotoxicity

**DOI:** 10.1038/cddis.2015.307

**Published:** 2015-10-22

**Authors:** A Gamir-Morralla, C López-Menéndez, S Ayuso-Dolado, G S Tejeda, J Montaner, A Rosell, T Iglesias, M Díaz-Guerra

**Affiliations:** 1 Department of Endocrine and Nervous System Physiopathology, Instituto de Investigaciones Biomédicas “Alberto Sols”, Consejo Superior de Investigaciones Científicas-Universidad Autónoma de Madrid (CSIC-UAM), Madrid 28029, Spain; 2 CIBERNED, Centro de Investigación Biomédica en Red sobre Enfermedades Neurodegenerativas, Instituto de Salud Carlos III, Madrid, Spain; 3 Neurovascular Research Laboratory, Institut de Recerca Vall d'Hebron, Neurovascular Unit, Department of Neurology, Universitat Autónoma de Barcelona, Barcelona, Spain

## Abstract

Kinase D-interacting substrate of 220 kDa (Kidins220), also known as ankyrin repeat-rich membrane spanning (ARMS), has a central role in the coordination of receptor crosstalk and the integration of signaling pathways essential for neuronal differentiation, survival and function. This protein is a shared downstream effector for neurotrophin- and ephrin-receptors signaling that also interacts with the *N*-methyl-d-aspartate type of glutamate receptors (NMDARs). Failures in neurotrophic support and glutamate signaling are involved in pathologies related to excitotoxicity and/or neurodegeneration, where different components of these dynamic protein complexes result altered by a combination of mechanisms. In the case of Kidins220/ARMS, overactivation of NMDARs in excitotoxicity and cerebral ischemia triggers its downregulation, which contributes to neuronal death. This key role in neuronal life/death decisions encouraged us to investigate Kidins220/ARMS as a novel therapeutic target for neuroprotection. As the main mechanism of Kidins220/ARMS downregulation in excitotoxicity is proteolysis by calpain, we decided to develop cell-penetrating peptides (CPPs) that could result in neuroprotection by interference of this processing. To this aim, we first analyzed in detail Kidins220/ARMS cleavage produced *in vitro* and *in vivo*, identifying a major calpain processing site in its C-terminal region (between amino acids 1669 and 1670) within a sequence motif highly conserved in vertebrates. Then, we designed a 25-amino acids CPP (Tat-K) containing a short Kidins220/ARMS sequence enclosing the identified calpain site (amino acids 1668–1681) fused to the HIV-1 Tat protein basic domain, able to confer membrane permeability to attached cargoes. Transduction of cortical neurons with Tat-K reduced Kidins220/ARMS calpain processing in a dose- and time-dependent manner upon excitotoxic damage and allowed preservation of the activity of pERK1/2 and pCREB, signaling molecules central to neuronal survival and functioning. Importantly, these effects were associated to a significant increase in neuronal viability. This Kidins220/ARMS-derived peptide merits further research to develop novel neuroprotective therapies for excitotoxicity-associated pathologies.

Neurotransmitters and neurotrophins regulate nervous system development, and preservation and remodeling of adult neural circuits. Prominent roles are played by glutamate, major excitatory neurotransmitter, and brain-derived neurotrophic factor (BDNF). By activating their receptors and signaling pathways, these ligands regulate multiple neuronal processes, including survival. Physiological stimulation of *N*-methyl-d-aspartate type of glutamate receptors (NMDARs) induces neuronal survival through extracellular signal-regulated kinases (ERKs) activation,^1^ antioxidant defenses induction,^2^ and cAMP response element-binding protein (CREB) phosphorylation.^3^ In turn, CREB increases expression of BDNF^4, 5^ and its receptor tropomyosin-related kinase B (TrkB).^6, 7^ Neurotrophin receptors enhance neuronal survival via signaling cascades involving PI3K-Akt, ERK,^8, 9^ CREB^10^ and nuclear factor kappa*-*B (NF-κB).^11^ Signaling requires large complexes formed at postsynaptic membranes by receptors and effectors. For instance, NMDAR association with ephrin receptor (Eph)B is critical for synaptic function,^12, 13^ while EphB activation by ephrin-B modulates NMDAR-dependent calcium influx and receptor expression.^14^ Likewise, TrkB interacts with ephrin-A7 and EphA.^15, 16^ In addition, Fyn tyrosine-kinase associates to TrkB and NMDAR-GluN2B,^17, 18^ while neural Shc (N-Shc), a neurotrophin signaling adaptor, also regulates NMDAR function.^19^ Coordination of these receptors crosstalk requires their shared effector Kinase D-interacting substrate of 220 kDa (Kidins220),^20^ also known as ankyrin repeat-rich membrane spanning (ARMS).^21^ Kidins220 bears twelve ankyrin repeats, four trans-membrane (TM) segments forming a KAP-NTPase domain,^20, 22^ a proline-rich region, a sterile alpha domain (SAM) and a PDZ ligand (PDZ-L) at the C-terminus.^20, 21^ It interacts with Trk receptors^21^ and is obligatory for neurotrophin-sustained ERK activation and neuronal differentiation.^23, 24, 25^ In addition, Kidins220 associates with NMDARs and modulates their ERK signaling and neuronal survival.^26^

Excitotoxicity is a specific neuronal-death process due to NMDAR overstimulation that contributes to neurodegeneration in acute disorders (ischemia, trauma and epilepsy) or chronic neurodegenerative diseases (Alzheimer's, Parkinson's or Huntington's).^27^ Defective neurotrophic support and signaling are also involved in neurodegeneration.^28, 29^ Altogether, these conditions cause high mortality and/or neurological impairment representing a social and health challenge. As there are no satisfactory treatments, it is capital to characterize excitotoxic mechanisms and how they affect proteins fundamental to survival/death choices^30^ in order to develop novel therapies. We discovered decreases in NMDAR^31, 32^ and TrkB^33^ signaling during excitotoxicity due to activation of the Ca^2+^-dependent protease calpain and transcriptional inhibition. Similar mechanisms control Kidins220 downregulation that contributes to neuronal death after NMDAR overactivation.^26^ Indeed, Kidins220 is crucial for neuronal survival as its knockdown decreases ERK activation and neuronal viability and enhances excitotoxic death.^26^ This key role in neuronal life/death decisions points to Kidins220 as a novel therapeutic target for neuroprotection. As calpain processing is the major mechanism of Kidins220 downregulation in excitotoxicity, we approach here the design of a neuroprotective peptide able to prevent Kidins220 calpain-dependent loss. First, we analyzed Kidins220 processing and identified a major calpain cleavage site within its C-terminus. We then designed a cell-penetrating peptide (CPP) containing the identified calpain site fused to a HIV-1 Tat protein basic domain, which confers membrane permeability and capability of crossing the blood–brain barrier (BBB).^34, 35^ Transduction of this peptide reduced Kidins220 calpain processing upon excitotoxicity and significantly increased neuronal viability.

## Results

### Kidins220 C-terminus is a major calpain target in excitotoxicity induced *in vitro* and *in vivo*

**Figure 1:**
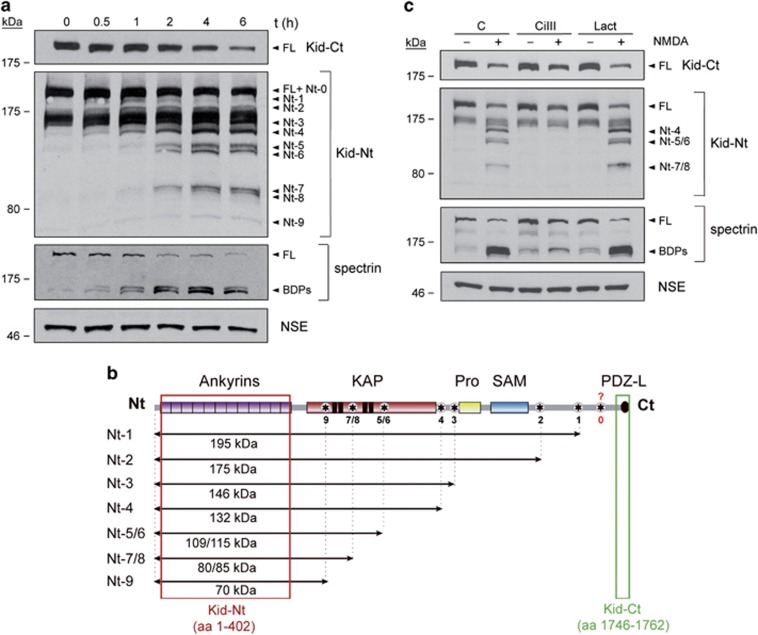
Approximate mapping of Kidins220 processing sites recognized by calpain after induction of excitotoxicity. (**a**) Immunoblot analysis of primary neuronal cultures (DIV 14) treated with high concentrations of NMDA (100 *μ*M) and glycine (10 *μ*M), herein indicated as NMDA, for 0–6 h. Kidins220 antibodies recognizing a C-terminal peptide (amino acids 1746–1762; Kid-Ct) or the N-terminal region (amino acids 1–402; Kid-Nt), or that specific for the protein neuronal-specific enolase (NSE) were used. Bands corresponding to endogenous full-length (FL) Kidins220 or its different processing N-terminal fragments (Nt-0/Nt-9) are indicated. Calpain activation was established by analyzing processing of FL spectrin (240 kDa) to characteristic breakdown products (BDPs; 145 and 150 kDa). (**b**) Schematic representation of Kidins220 protein domains with putative calpain targets. From the N-terminal (Nt) to the C-terminal (Ct), we observe twelve ankyrin repeats, a KAP-NTPase domain including four transmembrane sequences (TM1–TM4; black boxes), a proline-rich region (Pro), a sterile alpha motif (SAM) and a PDZ ligand (PDZ-L). The sequences recognized by Kidins220 antibodies are indicated by red (Kid-Nt) or green (Kid-Ct) boxes. Approximate location of at least nine calpain targets (asterisk), inferred from Kid-Nt immunoblot, with estimated molecular weight of corresponding fragments (labeled Nt-1/Nt-9) is indicated by horizontal arrows. The C-terminal Kidins220 region where a major calpain target is predicted (site 0) is indicated by a question mark. (**c**) Immunoblot analysis showing the effect of inhibitors specific for calpain (calpain inhibitor III, CiIII, 20 *μ*M) or the proteasome (lactacystin, Lact, 15 *μ*M) on Kidins220 calpain processing induced in excitotoxicity. Neuronal cultures were incubated with protease inhibitors for 1 h before addition of NMDAR co-agonists as above. Inhibitors were present for the duration of NMDA treatment (4 h). Spectrin analysis demonstrates the efficiency of calpain inhibition

To develop a neuroprotective strategy based on prevention of Kidins220 calpain processing induced by excitotoxicity, we first characterized cleavage topology and kinetics. We treated mature cortical neurons of 14 days *in vitro* (DIVs) with high concentrations of NMDAR co-agonists, NMDA and glycine, and analyzed Kidins220 by immunoblot using antibodies recognizing a C-terminal peptide (Kid-Ct)^20^ or the N-terminal region (Kid-Nt)^36^ (Figure 1a). As described,^26^ Kid-Ct showed a rapid decrease of full-length (FL) Kidins220 but no fragments, suggesting that those were very small and/or unstable. However, Kid-Nt exposed a slower FL-Kidins220 decay, suggesting a prominent calpain target close to the C-terminus (site 0, Figure 1b). Early cleavage at this sequence likely produced a relatively stable N-terminal fragment (Nt-0) difficult to resolve from FL-Kidins220 (Figure 1a). Thereafter, progressively smaller N-terminal fragments (Nt-1/Nt-9) emerged, allowing prediction of nine additional calpain targets within the KAP-NTPase domain and likely interdomain sequences (Figure 1b). Absence of complementary C-terminal fragments supported again the presence of a highly efficient calpain site nearby Kidins220 C-terminus that, once processed, avoided detection with Kid-Ct. Breakdown products (BDPs) from the widely used calpain-substrate spectrin confirmed calpain activation, while other neuronal proteins such as neuronal-specific enolase (NSE) were not significantly modified (Figure 1a). Requirement of calpain activation for Nt-1/Nt-9 production was corroborated by pre-incubation with calpain-specific inhibitor III (CiIII) (Figure 1c), which strongly prevented NMDA-induced processing in contrast to proteasome inhibitor lactacystin (Lact). Thus, an important mechanism of Kidins220 excitotoxic downregulation in cultured neurons is calpain processing of sequences downstream ankyrin repeats, being prominent a C-terminal target.

**Figure 2:**
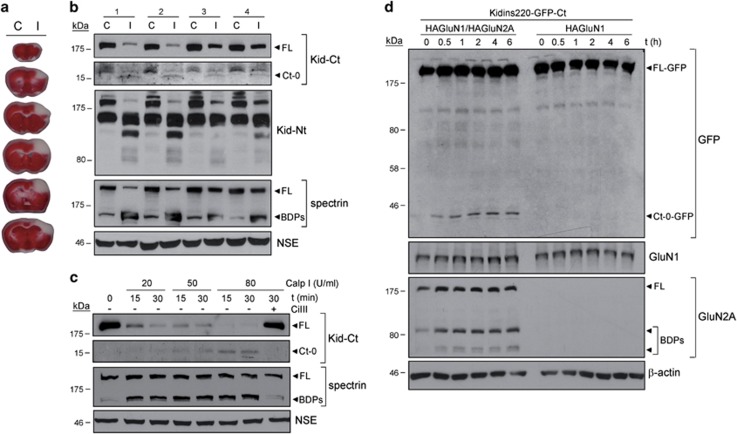
Kidins220 sequences approximately 15 kDa from the C-terminus are major calpain targets after calpain activation *in vivo* and *in vitro*. (**a**) Brain coronal sections of mice subjected to transient cerebral ischemia induced by dMCAO incubated with vital stain TTC showing a cortical infarct in the MCA territory of the ipsilateral hemisphere. MCA was compressed for 1 h followed by blood reperfusion for 24 h. (**b**) Analysis of Kidins220 processing after *in vivo* excitotoxicity induced by transient cerebral ischemia. Protein extracts were prepared from cortical infarcted regions (I) and corresponding areas of the contralateral hemisphere (C) of four mice subjected to dMCAO (1–4). Immnuoblot analysis of Kidins220 processing established a correlation between the degree of calpain activation, demonstrated by spectrin cleavage, the decrease of FL Kidins220, and the accumulation of different N-terminal intermediates (Nt-s) and a 15-kDa C-terminal fragment (Ct-0). The presence of this Ct-0 fragment, observed in longer exposures of Kid-Ct immunoblots, was higher in animals presenting a better calpain activation. (**c**) Protein extracts from cortical neurons subjected to *in vitro* digestion with purified calpain I (0, 20, 50 or 80 U/ml) for 15 or 30 min in the absence or presence of calpain inhibitor CiIII (20 *μ*M). The decrease of FL Kidins220 due to calpain activity correlated with the generation and accumulation of a major 15 kDa C-terminal fragment (Ct-0). (**d**) Analysis of Kidins220 C-terminal fragments produced in a heterologous system of excitotoxicity. To facilitate detection of C-terminal fragments, Kidins220 rat-coding sequence fused to GFP at its C-terminus (Kidins220-GFP-Ct) was expressed in HEK293T cells together with HA-tagged GluN1 and GluN2A, to produce a functional NMDAR, or GluN1 only, yielding an incomplete NMDAR. In HAGluN1/HAGluN2A-transfected cells stimulated with NMDA (0–6 h), GFP antibodies mainly detected FL Kidins220-GFP-Ct (FL-GFP) and its fragment Ct-0-GFP. In parallel, and in contrast to GluN1, subunit GluN2A (FL) was also processed and produced BDPs as expected for a calpain substrate. Neuronal-specific enolase (NSE) or *β*-actin levels were used as protein-loading controls

Next, it was important to analyze Kidins220 cleavage induced by *in vivo* excitotoxicity. We used a mice model of focal cerebral ischemia produced by distal occlusion of middle cerebral artery (dMCAO) where NMDAR overactivation is the major mechanism of neurodegeneration.^27^ MCA compression for 1 h followed by 24 h reperfusion induced large infarcts in cortical areas of this artery territory (Figure 2a).^37^ We compared Kidins220 levels in the infarcted and corresponding contralateral regions (average infarct volume 29.9±4 mm^3^; *n*=4 animals) observing an important decrease in FL-Kidins220 (Figure 2b). Kidins220 N-terminal fragments in ischemic tissue were similar to those of neurons subjected to excitotoxicity (see Figure 1a). However, differently from neurons, Kid-Ct identified a C-terminal fragment in infarcted tissue with an apparent molecular weight of 15 kDa (Ct-0). Relative levels of Ct-0 correlated with the degree of calpain activation, established by production of spectrin BDPs or Kidins220 N-terminal fragments (Figure 2b), confirming the importance of Kidins220 sequences nearby the C-terminus for *in vivo* calpain cleavage in ischemic brain. A similar Ct-0 fragment appeared in neuronal extracts after *in vitro* addition of purified calpain I (Figure 2c). The decrease of FL-Kidins220 and the corresponding accumulation of Ct-0 in a dose- and time-dependent manner resulted from calpain cleavage as they were inhibited by CiIII. *In vitro* digestion with calpain II produced similar results (Supplementary Figure 1). Finally, we confirmed that Ct-0 derived from Kidins220 as levels of this fragment decreased in parallel to those of FL after Kidins220 lentiviral silencing^26^ (Supplementary Figure 2). Altogether, these results demonstrate the existence of a calpain site nearby Kidins220 C-terminus that is efficiently processed *in vivo* after protease activation and produces a 15-kDa fragment of low stability.

To facilitate detection and isolation of Ct-0, we used a heterologous excitotoxicity system for ectopic expression of Kidins220-GFP-Ct and hypothesized that this approach might help fragment stabilization. HEK293T cells co-transfected with Kidins220-GFP-Ct and GluN1/GluN2A cDNAs, producing functional NMDARs, were stimulated with NMDA (Figure 2d). Anti-GFP antibodies showed limited FL-Kidins220-GFP-Ct (FL-GFP) downregulation. However, an abundant short fragment (Ct-0-GFP) was specifically induced from early times upon NMDA treatment of GluN1/GluN2A-transfected cells but absent in cells expressing an incomplete NMDAR. The molecular weight of Ct-0-GFP (37 kDa) was compatible with a fusion of GFP (26.9 kDa) and a 10-kDa Ct-0 fragment. The discrepancy between Ct-0 deduced size or that found in ischemic brain and neuronal extracts digested *in vitro* could be due to post-translational modifications absent in HEK293 cells or simply an effect of GFP fusion over Ct-0 electrophoretic properties. Subunit-specific antibodies probed expression of GluN1 and GluN2A and NMDA-dependent processing of GluN2A, as expected.^32^ Thus, the modest excitotoxic response observed in HEK293 cells *versus* neurons might contribute together with GFP fusion to stabilize Kidins220 C-terminal fragments.

### Identification of an evolutionary conserved sequence motif for calpain cleavage within Kidins220 sequence

**Figure 3:**
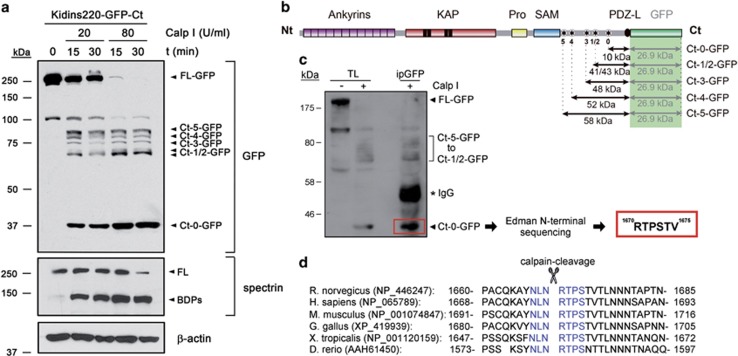
Establishment of the major calpain-recognition sequence in Kidins220 C-terminus. (**a**) Immunoblot analysis of protein extracts obtained from HEK293T cells expressing Kidins220-GFP-Ct subjected to *in vitro* digestion with purified calpain I (20 or 80 U/ml) for 15 or 30 min. The GFP antibody reveals processing of FL Kidins220-GFP-Ct (FL-GFP) and the corresponding accumulation of a major C-terminal GFP-fused fragment (Ct-0-GFP) together with low amounts of additional fragments (Ct-1-GFP/Ct-5-GFP). Spectrin processing and levels of *β*-actin were used as controls of calpain activity and protein loading, respectively. (**b**) Schematic representation of fusion protein Kidins220-GFP-Ct with putative minor (Ct-1-GFP/Ct-5-GFP) and major (Ct-0-GFP) calpain targets (asterisks), their approximate location being inferred from immunoblots with antibodies specific for GFP. The fragments produced, together with their estimated molecular weights, are indicated by horizontal arrows. The contribution of GFP to the molecular weight of those fragments is depicted separately (green box). (**c**) Purification of Ct-0-GFP and Edman sequencing. Total lysates (TL) from HEK293T cells transfected with Kidins220-GFP-Ct were left undigested or subjected to a preparative digestion with purified calpain (80 U/ml). Digested lysates were then immunoprecipitated (IP) with GFP antibodies. Small fractions of total lysates or immunoprecipitates were analyzed with GFP antibodies to corroborate the accumulation and concentration of Ct-0-GFP fragment (highlighted by a red box). The band corresponding to this fragment was cut from a preparative filter stained with Coomasie Blue and subjected to Edman degradation. The N-terminal sequence obtained and the amino acids position (1670–1675) in rat protein (NP_446247) are shown below. (**d**) Comparison of rat Kidins220 region containing the major calpain cleavage site identified above to homologous regions in five additional vertebrate species, human (NP_065789), mice (NP_001074847), chicken (XP_419939), frog (NP_001120159) and zebrafish (AAH61450). A high level of sequence conservation is observed in all vertebrate species analyzed and, particularly, a seven amino acids stretch comprising Kidins220 calpain-processing site 0 (blue) is completely conserved

To design a neuroprotective strategy to interfere Kidins220 excitotoxic processing, it was crucial to identify the major C-terminal sequence early cleaved by calpain. Accurate *in silico* prediction of Kidins220 calpain sites is difficult as structural bases for calpain-substrate recognition are not well defined^38^ and no consensus sequences exist. Therefore, we decided to affinity purify Kidins220 Ct-0 fragment and sequence its N terminus by Edman degradation to identify this cleavage site. Because *in vitro* calpain processing (Figure 2c) or GFP fusion (Figure 2d) apparently increased fragment stability, we incubated extracts from HEK293T cells expressing Kidins220-GFP-Ct with calpain I (Figure 3a). Immunoblotting revealed FL-Kidins220-GFP dose- and time-dependent processing and consequent Ct-0-GFP accumulation. We also detected minor processing intermediates (Ct-1-GFP/Ct-5-GFP, Figure 3b) that might be complementary to some N-terminal fragments (Figure 1) and stabilized by GFP fusion. Furthermore, while Kid-Ct showed similar efficiencies of calpain processing for endogenous or Kidins220-GFP-Ct, higher levels of GFP-Ct-fused polypeptides accumulated compared with endogenous fragments (Supplementary Figure 3). Next, we isolated Ct-0-GFP by *in vitro* calpain digestion of lysates from Kidins220-GFP-Ct-transfected HEK293T cells and GFP immunoprecipitation (Supplementary Figure 4). We confirmed the efficiency of Ct-0-GFP immunoprecipitation, the major protein within GFP immunocomplexes (Figure 3c). Edman sequencing of purified Ct-0-GFP (Supplementary Figure 4) identified sequence RTPSTV, which corresponded to rat Kidins220 amino acids 1670–1675 (Figures 3c and d). This result identified calpain cleavage between amino acids 1669 and 1670 to produce a C-terminal fragment of predicted molecular weight 10.2 kDa. This cleavage site was completely conserved in human, mice, chicken, frog and zebrafish (Figure 3d). Furthermore, surrounding sequences were also highly conserved, supporting the importance of this calpain target for Kidins220 downregulation in excitotoxicity.

### A CPP containing amino acids 1668–1681 inhibits Kidins220 calpain-dependent downregulation

**Figure 4:**
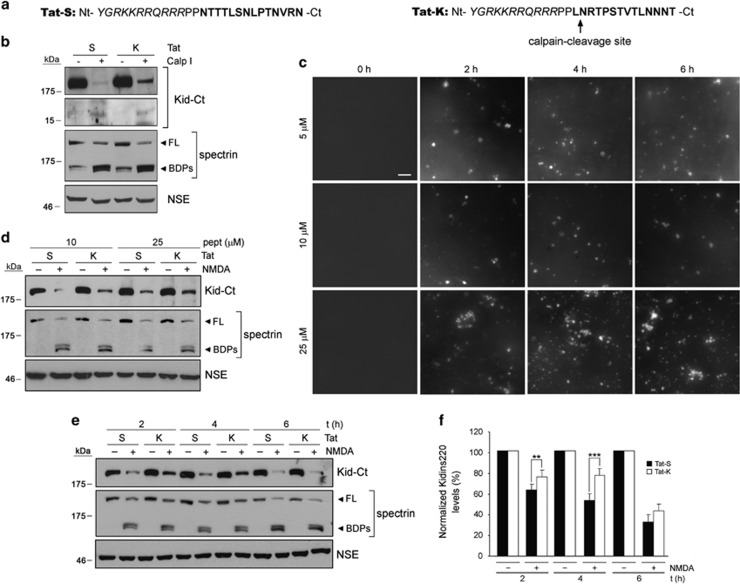
Design of a CPP able to specifically inhibit Kidins220 calpain processing induced by excitotoxicity. (**a**) Cell-membrane peptide Tat-K contains from the N- to the C-terminus amino acids 47–57 of the HIV-1 Tat protein (italic) followed by two proline linker residues and rat Kidins220 amino acids 1668–1681 (bold), which include the major calpain cleavage site (arrow). Control peptide Tat-S is similar to Tat-K but Kidins220 amino acids (bold) are randomly organized. (**b**) Analysis of Tat-K interference of *in vitro* calpain processing of Kidins220. Protein extracts from cortical neurons were subjected to *in vitro* digestion with purified calpain I (20 U/ml) for 15 min in the presence of peptides Tat-K (K) or Tat-S (S) (0.5 *μ*M). Results were analyzed by immunoblot with Kidins220 (Kid-Ct), spectrin and NSE antibodies. (**c**) Establishment of optimum conditions for peptide entry into cortical neurons. Primary cultures of neurons (DIV 14) were incubated with 5, 10 or 25 *μ*M of FITC-labeled control peptide (FITC-Tat-S) for 2, 4 or 6 h and pictures were taken in a conventional fluorescence microscope. Scale bar, 25 *μ*m. (**d**) Dose response of Tat-K-specific interference of Kidins220 calpain processing induced by NMDAR overactivation. Cortical neurons were pre-incubated for 1 h with 10 or 25 *μ*M of Tat-K (K) or Tat-S (S) before NMDA addition for 4 h and analyzed by immunoblot with Kidins220 (Kid-Ct), spectrin and NSE antibodies. (**e**) Time course of Tat-K-specific interference of Kidins220 calpain processing induced by excitotoxicity. Neurons were pre-incubated for 1 h with 25 *μ*M of Tat-K (K) or Tat-S (S) before NMDA addition for 2, 4 or 6 h and analyzed as before. (**f**) Quantitation of Tat-K protection of Kidins220 processing induced by excitotoxicity. Levels of Kidins220 were established by densitometric analysis of immunoblots using NIH Image software and normalized to those of NSE present in the same samples. For each time point, Kidins220 levels in neurons treated with NMDA in the presence of Tat-S or Tat-K are represented as relative values to those obtained in cultures incubated with the same peptide but no NMDA, arbitrarily given a 100% value. Average of five independent experiments with S.E.M. is given. Statistical significance was determined by paired Student's *t*-test (**P<0.01, ***P<0.001)

Next, we designed a 27-amino acids CPP containing residues 47–57 of HIV-1 Tat protein fused to Kidins220 amino acids 1668–1681 (Figure 4a, Tat-K) to interfere Kidins220 calpain recognition and excitotoxic processing. We also synthesized a control-scrambled peptide where Kidins220 amino acids were randomly organized (Figure 4a, Tat-S). First, we assayed Tat-K efficacy to prevent Kidins220 *in vitro* calpain processing (Figure 4b). Neuronal extracts incubated with calpain I and Tat-K showed less Ct-0 and increased FL-Kidins220 resistance to processing compared with those containing Tat-S. Thus, Tat-K could prevent Kidins220 *in vitro* calpain degradation. Then, we confirmed peptide membrane permeability and established optimal entry conditions. FITC-labeled Tat-S (FITC-Tat-S) entered neurons in a dose-dependent manner, the best results obtained for 10–25 *μ*M (Figure 4c). Intensity of internalized fluorescence was similar at the different incubation times, suggesting relative peptide stability once inside cells. Appropriate conditions for peptide pre-incubation before induction of excitotoxicity were also set by studying early kinetics of peptide delivery, observing the highest fluorescence levels after 1 h (Supplementary Figure 5). Finally, we analyzed Tat-K ability to reduce Kidins220 calpain processing induced by NMDAR overactivation. Neurons were pre-incubated for 1 h with Tat-K or Tat-S (10 or 25 *μ*M) before NMDA addition (Figure 4d). A strong decrease in Kidins220 levels was concomitant to calpain activation in Tat-S-treated neurons while that protein was better preserved by Tat-K, with no apparent effect on spectrin. To further characterize Tat-K actions, neurons were pre-incubated with Tat-S or Tat-K (25 *μ*M) and NMDA treated for different times (Figure 4e). In the presence of Tat-S, NMDA induced a strong decrease in Kidins220 from early times (2 h) of excitotoxicity, an effect increased later on. In contrast, pre-treatment with Tat-K significantly reduced Kidins220 downregulation at 2 or 4 h of NMDA stimulation (Figure 4e). Quantitation showed that Kidins220 levels remained significantly higher after 4 h of NMDA incubation in Tat-K (78±7%) compared with Tat-S-treated neurons (53±6% *P<*0.001; *n*=6; Figure 4f). This response was not due to changes in Kidins220 basal levels induced by peptide pre-incubation as they did not occur in non-excitotoxic conditions (Supplementary Figure 6). We also analyzed Tat-K effects on excitotoxic cleavage of other calpain substrates that associate to Kidins220 and are important for NMDAR signaling (Supplementary Figure 7). Pre-incubation with Tat-K marginally preserved NMDA-induced processing of components of the NMDAR complex such as GluN2A and GluN2B^26^ subunits or their interacting protein PSD95, while had no effect on spectrin. These interferences might also have important consequences for neuroprotection as all these proteins are central to neuronal survival and functioning. In conclusion, these results demonstrate that Tat-K, a CPP containing a major Kidins220 calpain-cleavage site, is able to efficiently hinder processing of this protein at early times of excitotoxicity.

### Tat-K transduction confers neuroprotection and preserves ERK1/2 and CREB survival pathways

**Figure 5:**
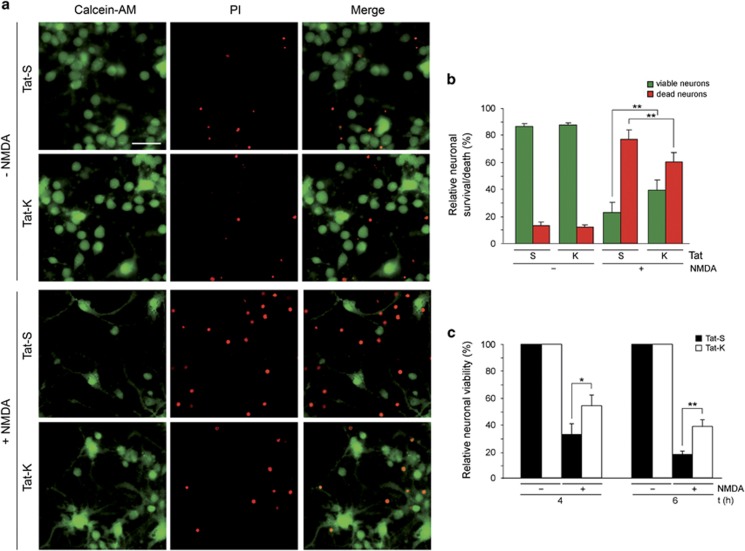
Neuroprotective effect exerted by Tat-K transduction on excitotoxic neuronal death. (**a**) Effect of Tat-K on cell death induced by NMDAR overactivation. Primary cultures of cortical neurons (DIV 14) were pre-incubated for 1 h with Tat-S or Tat-K (25 *μ*M) and treated with NMDA for 4 h or left untreated. Next, viable and dead cells were visualized by simultaneous fluorescence staining with calcein-AM (1 *μ*M; green) and propidium iodide (PI; 0.5 mM; red), respectively, for 15 min at 37 º C in the dark. Representative images for each condition are shown. Scale bar, 25 *μ*m. (**b**) Quantitation of Tat-K effect on the relative percentage of viable/dead neurons found in excitotoxic conditions. A minimum of five random fields containing at least 500 neurons each were counted per experimental condition. The percentage of viable (green bars) *versus* death (red bars) neurons was expressed relative to the total number of neurons examined, arbitrarily given a 100% value. Average of seven independent experiments with S.E.M. is represented. Statistical significance was determined by paired Student's *t*-test (**P<0.01). (**c**) Quantitation of the effect of Tat-K on neuronal viability after induction of excitotoxicity. Cultures of cortical neurons were pre-incubated for 1 h with Tat-S or Tat-K (25 *μ*M) and treated with NMDA for 4 or 6 h as before or left untreated. Neuronal viability was established by MTT reduction assay after subtracting the contribution of the glial cells present in the mixed cultures to absorbance as indicated in the Methods section. For each time point, viability of neurons treated with NMDA in the presence of Tat-S or Tat-K is represented relative to that of neurons incubated with the same peptide but no NMDA, arbitrarily given a 100% value. Average of five independent experiments with S.E.M. is given. Statistical significance was determined by paired Student's *t*-test (*P<0.05, **P<0.01)

We next explored if inhibition of Kidins220 calpain processing by Tat-K could neuroprotect from excitotoxicity and, in that case, establish the protective mechanism. Excitotoxicity was induced in neurons pre-incubated with CPPs followed by fluorescence staining of viable and dead cells with calcein-AM and propidium iodide (PI), respectively (Figures 5a and b). In neurons pre-incubated with Tat-S, NMDA treatment provoked a strong decrease in viability and a correlative increase in death compared with untreated cultures (Figure 5a). However, the number of viable neurons in Tat-K-transduced NMDA-treated cultures almost doubled that obtained in Tat-S-incubated cells (40±7% *versus* 23±7%, respectively, *P*<0.01; *n*=7; Figure 5b). Accordingly, the percentage of dead neurons was also statistically different (60±7% *versus* 77±7%, *P*<0.01). The neuroprotective effect of Tat-K after NMDAR overstimulation was also analyzed by MTT assays (Figure 5c). The viability of neurons incubated with NMDA for 6 h in the presence of Tat-S was only 17±4% relative to non-stimulated cells while survival increased to 41±6% (*P*<0.05; *n*=5) in Tat-K-treated neurons. These peptides had no toxicity as no significant differences in viability were found in transduced *versus* non-transduced neurons (Supplementary Figure 8). Our results prove a clear neuroprotective effect of Tat-K on excitotoxicity that correlates with this peptide ability to interfere Kidins220 calpain processing.

**Figure 6:**
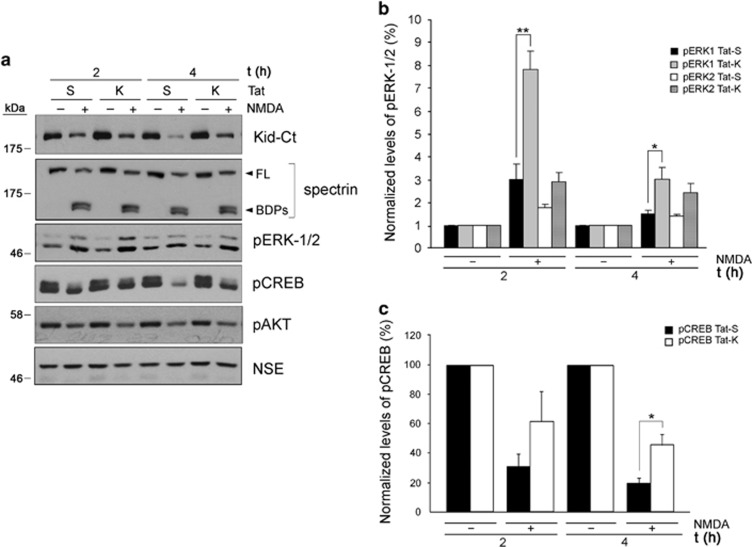
ERK1/2 and CREB survival pathways are preserved in excitotoxic conditions in neurons treated with Tat-K. (**a**) Effect of Tat-K on ERK1/2, AKT and CREB activation induced by NMDAR overactivation. Neurons were pre-incubated for 1 h with 25 *μ*M of Tat-K (K) or Tat-S (S) before NMDA addition for 2 or 4 h and analyzed by immunoblot with phospho-specific antibodies recognizing the active forms of ERK1/2 (pERK1/2, Thr^202^/Tyr^204^), AKT (pAKT, Ser^473^) and CREB (pCREB, Ser^133^). (**b**) Quantitation of Tat-K effect on pERK1/2 levels. Levels of pERK1 and pERK2 were established by densitometric analysis of immunoblots using NIH Image software and normalized to those of NSE present in the same samples. For each time point, pERK1 or pERK2 levels in neurons treated with NMDA in the presence of Tat-S or Tat-K were represented as fold increase compared with those obtained in cultures incubated with the same peptide but no NMDA, arbitrarily given a value of one. Average of five independent experiments with S.E.M. is given. Statistical significance was determined by paired Student's *t*-test (*P<0.05, **P<0.01). (**c**) Quantitation of Tat-K effect on CREB activation. Levels of pCREB were established and normalized as before, and represented as percentage of those obtained in cultures incubated with the same peptide but no NMDA, arbitrarily given a 100% value. Average of three independent experiments with S.E.M. is given. Statistical significance was determined by paired Student's *t*-test (*P<0.05, **P<0.01)

Maintenance of higher Kidins220 levels during excitotoxicity by Tat-K treatment might allow preservation of important neuronal survival pathways where this protein participates. In neurons, Kidins220 silencing decreases basal ERK1/2 activity, alters ERK1/2 activation induced by NMDAR overstimulation and reduces neuronal survival.^26^ Therefore, calpain-induced Kidins220 downregulation might significantly contribute to excitotoxic neuronal death affecting this pathway. We analyzed ERK1/2 activation in cultures pre-incubated with Tat-K or Tat-S and treated with NMDA for 2 h, time of maximum kinase activation,^26^ or 4 h (Figures 6a and b). As in control neurons, NMDA induced a transient pERK1/2 augmentation in Tat-S and Tat-K pre-treated cultures (Figures 6a and b). However, ERK1/2 activation was enhanced by Tat-K and correlated with inhibition of Kidins220 processing. Compared with control neurons, 2 h of NMDA stimulation augmented pERK1 by a factor of 8±1 in Tat-K-treated cultures, value significantly higher than that obtained with Tat-S (3±1, *P*<0.01; *n*=5) (Figure 6b). NMDAR overactivation also induces a dominant CREB shut-off that affects neuronal survival.^3^ Thus, we also analyzed CREB activity in these experimental conditions and found that Tat-K treatment before excitotoxicity also rendered significant increases in active pCREB (Figures 6a and c). Importantly, PI3K/Akt survival pathway, which is also deactivated by excitotoxicity,^39^ was unaffected by Tat-K as determined detecting active pAkt (Figure 6a).

In conclusion, our results unveil a novel strategy for neuroprotection based on the use of Tat-K, and strongly support that this peptide, by hindering Kidins220 calpain processing induced by excitotoxicity, is able to decrease neuronal death and specifically enhance ERK1/2 and CREB activity, preserving signaling pathways central to neuronal survival.

## Discussion

Herein we have discovered a major Kidins220 calpain cleavage site highly conserved in vertebrates. On the basis of this sequence, we have developed a neuroprotective peptide that decreases Kidins220 calpain processing and excitotoxic death in cortical neurons, establishing this sequence motif as a novel target for neuroprotection in excitotoxicity-associated pathologies. Analysis of Kidins220 cleavage in excitotoxic neurons using a N-terminal antibody (Kid-Nt) allowed prediction of at least nine calpain targets downstream ankyrin repeats. Progressive accumulation of shorter intermediates along NMDA treatment suggested a similar processing efficiency for all of them. However, an antibody recognizing Kidins220 last 17 amino acids (Kid-Ct) could not detect fragments in excitotoxic neurons suggesting a nearby and prominent calpain-cleavage site, and the production of a low-stability fragment. Supporting this hypothesis, brain tissue from ischemic mice rendered a 15-kDa C-terminal Kidins220 fragment, which could be also observed by *in vitro* digestion of neuronal lysates with calpain I or II. The lower stability of this fragment in the cellular model of excitotoxicity might be simply related to a higher activity, compared with cells *in vivo,* of additional proteases further degrading the 15-kDa fragment. Anyway, appearance of this fragment in ischemic brain supported the relevance of pursuing the identification of that specific calpain-cleavage site within Kidins220 to develop a neuroprotective strategy.

The pre-eminence of this calpain site at the very C-terminal region indicates that Kidins220 PDZ-L will be rapidly lost in excitotoxicity having important functional consequences. This PDZ-L is responsible for Kidins220 localization at the neuromuscular junction through association with *α*-syntrophin, a PDZ protein enhancing EphA4 signaling in a Kidins220-dependent manner^40^ and known to associate with NMDAR-GluN2A-C subunits.^41^ In addition, Kidins220 PDZ-L interacts with the PDZ protein S-SCAM forming a tetrameric complex with PDZ-GEF1 and Trk,^25^ a major component for neurotrophin-induced sustained ERK stimulation leading to differentiation. Therefore, Kidins220 PDZ-L cleavage will rapidly uncouple this effector from these and other yet unknown downstream signaling pathways and hamper functions mediated by this motif. Some minor Kidins220 calpain sites might be located at KAP-NTPase domain bearing the four TM segments.^20^ Other NTPases in this KAP family have been suggested to participate in assembling/disassembling of signaling complexes mostly associated to the cytosolic side of cell membranes in a NTPase-dependent way.^22^ Independently of Kidins220 participation in similar functions, processing of KAP-NTPase domain would primarily affect Kidins220 putative intrinsic enzymatic activity. In addition, as TM4 mediates Kidins220/Trk interaction,^23^ calpain cleavage at this domain might impact its role as neurotrophin effector. The remaining processing sites could occur in Kidins220 interdomain sequences, a frequent trend among calpain substrates.^42^

Because *in vitro* excitotoxicity produced a highly unstable Kidins220 C-terminal fragment, we obtained a more stable GFP-fused fragment by *in vitro* calpain processing of Kidins220-GFP-Ct. By purifying this fragment, we identified the precise calpain cleavage site between amino acids 1669 and 1670 within rat Kidins220 sequence. Mechanisms of calpain substrate recognition and cleavage are largely unknown^38^ and there is no consensus recognition sequence. Different bioinformatic tools allow *in silico* predictions based on empirically derived rules for position-based residues preference (Position-Specific Scoring Matrix Methods, PSSM), Support Vector Machine (SVM) algorithms for machine learning (Linear or Radial Basis Function Kernel, Multiple Kernel Learning),^43^ or a Group-Based Prediction System (GPS) algorithm.^44^ We analyzed Kidins220 C-terminal 1000 amino acids using these tools (available at http://www.calpain.org and http://ccd.biocuckoo.org) and found a coincident hit between amino acids 1669–1670, the site identified by Edman sequencing. The resulting 93-amino-acids polypeptide bears a destabilizing N-terminal arginine that might be a target for the Arg/N-end rule pathway and explain its short life.^45^ Interestingly, seven amino acids contained within this processing site are completely conserved in six different vertebrate species, including humans, supporting the importance of this sequence for Kidins220 downregulation in excitotoxicity.

Calpain activation regulates neuronal death in brain damage and neurodegeneration^46, 47^ and, therefore, pharmacological inhibitors have been considered as potential neuroprotective drugs.^48, 49^ However, as this protease is likewise required for normal neuronal function, calpain inhibition promotes neuronal survival after injury but also causes deficits in spine density and dendritic branching complexity associated with impaired LTP and spatial memory.^50^ The use of peptides to interfere calpain pathological activities on specific substrates is emerging as an alternative to generic inhibitors. It has already succeeded in blocking NMDA-induced truncation of mGluR1*α*^51^ or transient-receptor potential canonical 6 (TRPC6) channel,^52^ proteins having neuroprotective roles. We have designed a peptide that inhibits Kidins220 excitotoxic calpain downregulation and tested if stabilization of this protein, central to neuronal physiology (reviewed in Neubrand *et al.*^53^), resulted in neuroprotection. This peptide, Tat-K, bearing the identified calpain-recognition sequence (amino acids 1668–1681), efficiently inhibits early NMDA-induced Kidins220 processing and exhibits neuroprotection against *in vitro* excitotoxicity. The decrease of Tat-K efficacy after 4 h of NMDAR overactivation is probably consequence of intrinsic peptide instability and/or inactivation by calpain processing once inside neurons. Anyway, these neuroprotective effects could be still sufficient *in vivo* to rescue neurons of the ischemic penumbra from secondary death induced by dying cells in the infarct core.

Tat-K-induced neuroprotection correlated with specific preservation of ERK1/2 and CREB activities, both central to neuronal survival. Excitotoxic regulation of ERK activity highly depends on experimental conditions. In our model, where both synaptic and extrasynaptic NMDARs are overstimulated and downregulated, we demonstrated an early activation of ERK peaking at 2 h,^26^ which is probably induced by the synaptic pool of receptors, followed by a gradual shut-off possibly derived from NMDAR downregulation.^31, 32^ Kidins220 silencing hampers sustained neurotrophin-induced ERK1/2 activation,^23, 24^ decreases basal and NMDA-dependent ERK1/2 activity and neuronal viability, and enhances excitotoxic death.^26^ Thus, inhibition of Kidins220 processing by Tat-K might help to preserve ERK1/2 signaling, which mediates synaptic NMDAR-dependent neuronal plasticity and survival.^1^ In addition, CREB activity preservation in excitotoxicity by Tat-K would be highly relevant to neuronal viability and function because this transcription factor is involved in activity-dependent^3^ or neurotrophin-mediated^10^ neuronal plasticity and survival. A secondary mechanism induced by Tat-K might also have a minor contribution to maintenance of ERK1/2 and CREB activities. Unlike generic calpain inhibitors, Tat-K did not have a general effect on excitotoxicity-induced processing of alternative calpain substrates such as spectrin. However, a small Tat-K effect was observed on stability of other Kidins220-associated proteins undergoing calpain cleavage during excitotoxicity such as NMDAR-GluN2-subunits^26^ or PSD95. This fact supports the existance of an indirect mechanism, probably derived from Kidins220 preservation, responsible of partial stabilization of NMDAR complexes. As these complexes are central to neuronal survival and function, this secondary mechanism of Tat-K action could be also important for neuroprotection.

CPP-based strategies similar to the one described here have shown a great potential for the treatment of acute and chronic disorders of the nervous system,^54^ mostly due to their low toxicity and capability to cross cell membranes and BBB.^34, 35^ Results of a phase-2 trial in patients undergoing endovascular aneurysm repair showed a decrease in the number of ischemic lesions typically induced by surgery after administration of Tat-NR2B9c.^55^ This CPP contains NMDAR–GluN2B–PDZ-L and dissociates a ternary complex formed with PSD95 and nNOS, uncoupling NMDARs from neurotoxic signaling^56^ and reducing neuronal damage in ischemia models without affecting synaptic activity.^56, 57^ Additional therapeutic strategies aimed to prevent downregulation of proteins that, like Kidins220, are important to neuronal survival have a great potential for neuroprotection in excitotoxicity-associated pathologies.

## Materials and Methods

### Materials and chemicals

NMDA, glycine, cytosine *ß*-d-arabinofuranoside (AraC), poly-l-lysine, l-laminin and propidium iodide were acquired from Sigma Co. (St. Louis, MO, USA). Calpain I (*μ*-calpain), calpain II (m-calpain), carbobenzoxy–valinyl–phenylalaninal (CiIII) and lactacystin (Lact) were from Calbiochem–Merck Bioscience (Darmstadt, Germany), and antagonist 2-amino-phosphopentanoic acid (DL-AP5) from Tocris (Bristol, UK). Lipofectamine 2000, Glutamax and Calcein-AM were purchased from Invitrogen-Live Technologies (Carlsbad, CA, USA). ECL Western Lighting Chemiluminisence Reagent Plus was from Perkin-Elmer Life Sciences (Boston, MA, USA) and BCA reagent was from Pierce Thermo Scientific (Rockford, IL, USA). Tat-S, FITC-Tat-S and Tat-K peptides were obtained from Immunostep (Salamanca, Spain).

### Antibodies

Rabbit polyclonal antibodies recognizing N-terminal (Kid-Nt)^36^ (generously provided by Dr. G. Schiavo) or C-terminal (Kid-Ct)^20^ regions of Kidins220 were previously described. Rabbit polyclonal antibodies against active phospho-p44/p42 MAPK (Thr^202^/Tyr^204^), herein pERK1/2, and phospho-Akt kinase (Ser^473^) were from Cell Signaling Technology (Beverly, MA, USA) while those for active phospho-CREB (Ser^133^) and NSE were from Millipore Corporation (Billerica, MA, USA) and ICN Biomedicals (Costa Mesa, CA, USA), respectively. Mouse monoclonal antibodies against *ß*-actin, GluN2B and GluN1 were purchased from Sigma Co., BD Biosciences (San Jose, CA, USA) and Pharmigen (San Diego, CA, USA), respectively. Mouse monoclonal antibodies recognizing non-erythroid spectrin and PSD95 were from Chemicon (Temacula, CA, USA). Mouse monoclonal and rabbit polyclonal antibodies against GFP were obtained from Invitrogen-Live Technologies. Goat and rabbit polyclonal antibodies recognizing, respectively, GluN2A C-terminus and p44/p42 MAPK (herein total ERK1/2) were purchased from Santa Cruz Biotechnology (Santa Cruz, CA, USA). Horseradish peroxidase-conjugated antibodies were from General Electric (Fairfield, CT, USA).

### Animal model of transient cerebral ischemia

All animal procedures were performed in compliance with Spanish legislation and European Union directives and approved by the local Animal Care Committee (protocol number 48/13, Vall d'Hebron Research Institute). Adult male Balb/c mice (25–30 g; Charles River Laboratories, Cerdanyola, Spain) were given free access to food and water before surgery, performed as previously described.^37^ Mice were anesthesized with isoflurane (4% for induction, 2% for maintenance in air, 79% N_2_ : 21% O_2_; Abbot Laboratories, Madrid, Spain). Body temperature was maintained at 36.5–37 °C using a self-regulating heating blanket and a rectal probe. An incision was made between the left eye and ear under an operating microscope (Leica MS5; Leica, Heerburg, Switzerland) and the temporal muscle divided, exposing the skull lateral aspect. The MCA was identified through the semi-translucent skull, and a small burr hole (2–3-mm diameter) was made with a microdrill at the level of the inferior cerebral vein to expose the M1 portion, leaving the dura intact. Drying injury of the tissue was prevented by continuous hydration via saline application to the area. Regional cerebral blood flow (CBF) was recorded from 5 min before MCAO to 5 min after reperfusion by laser Doppler flowmetry using a flexible fibreoptic (0.5-mm diameter; Moor Instruments, Devon, UK) placed on the surface of the M1 parietal branch bifurcation. The MCA was directly compressed using a micromanipulator holding a 30G blunted needle (0.4-mm diameter). After 60 min of occlusion, the needle was carefully removed and the blood flow restored. Arterial occlusion was considered successful when CBF was maintained for 60 min below 20% of baseline value, and reperfusion if CBF recovered to at least a 75% of baseline values. Twenty-four hours after blood reperfusion, animals were killed and their brains sectioned into 1-mm-thick coronal slices and briefly stained with a 2% solution of triphenyltetrazolium chloride (TTC; Merck Bioscience, Darmstadt, Germany) in the cold to avoid endogenous post-mortem calpain activation. The unstained area of the cerebral cortex, defined as infarcted tissue, as well as the corresponding region in the contralateral hemisphere were dissected to prepare protein lysates, frozen and stored at −80 °C until use.

### Primary culture and treatment of cortical neurons

Neuronal cultures were prepared from cerebral cortex of 19-day-old Wistar rat embryos as we have previously described.^26, 31^ Rats were obtained from the animal care facility at the Instituto de Investigaciones Biomedicas “Alberto Sols” (CSIC-UAM, Madrid, Spain). Animal procedures were approved by the CSIC ethical committee and performed in compliance with European Directive 2010/63/EU. Neurons were used after 14 days *in vitro* (DIVs) and pre-treated or treated for different times, as indicated, with the following concentrations of reactives: 100 *μ*M NMDA, 10 *μ*M glycine, 20 *μ*M CiIII, 15 *μ*M lactacystin and 5, 10 or 25 *μ*M Tat-S or Tat-K. Excitotoxicity was induced by overstimulation of cortical neurons with NMDAR co-agonists NMDA and glycine (herein denoted NMDA). Unless otherwise stated, inhibitors and Tat-peptides were added 1 h before NMDA treatment and remained in the culture media for the duration of the experiment. When indicated, FITC-conjugated Tat-S was used, and its entrance into cultured neurons was followed by *in vivo* fluorescent microscopy using an Eclipse TE2000-U Nikon microscope and a Hamamatsu digital camera C10600. Pictures were processed with NIS-Elements BR 3.00, SP6 (Nikon Laboratory Imaging, Prague, Czech Republic) and Adobe CS3 Extended software (Adobe Systems Inc., San Jose, CA, USA).

### cDNA constructs

Plasmid driving the expression of N-terminal tagged rat HA-GluN2A subunit has been previously described^32^ and contains a hemagglutinin (HA) epitope between residues 51 and 52 of this protein. To obtain plasmid Syn-HA-GluN1, a BamHI fragment isolated from pME18S-HA-GluN1 (a generous gift from Dr. T. Yamamoto, University of Tokyo, Tokyo, Japan^17^) was subcloned into the Syn-GFP lentiviral plasmid^32^ digested as before. This plasmid produces a GluN1 subunit with an HA epitope between amino acids 27 and 28. The vector Kidins220-GFP-Ct encoding rat Kidins220 fused at the C-terminal with GFP has been previously used,^58^ and was generated by subcloning a SmaI/NdeI fragment containing FL rat Kidins220 cDNA from pGEMT easy (Promega Corporation, Madison, WI, USA) into pEGFP-N2 SmaI site.

### Culture and transfection of HEK293T-cells

Human embryonic kidney 293T cells (HEK293T) were cultured at 37 °C in Dulbecco's modified Eagle's medium (Invitrogen-Live Technologies), supplemented with 10% fetal bovine serum, 2 mM glutamine and 100 U/ml of penicillin/streptomycin in an humidified atmosphere containing 5% CO_2_. Transfection was performed in serum-free medium with Lipofectamine 2000 reagent and, 4 h after addition, liposomes were removed and cells fed with supplemented medium and maintained in culture for 48 h. In those experiments where HA-GluN1 and HA-GluN2A subunits were co-expressed with Kidins220-GFP-Ct, the NMDAR competitive antagonist DL-AP5 (2 mM) was added after transfection to prevent HEK293T excitotoxic death by NMDAR activation due to glutamate present in the culture medium.^59^ Removal of the antagonist was performed just before NMDA stimulation.

### Lentiviral infection of neuronal cultures

Plasmids and lentivirus suspensions were prepared as previously described.^26^ Neurons grown for eight DIVs were transduced with lentivirus expressing *Kidins220* shRNA (shK) or a control shRNA (shC) directly added to the growing media and infection proceeded for six additional days.

### Preparation of protein extracts and immunoblot analysis

Protein extracts from primary cultures, HEK293T or brain tissue were prepared as described.^26^ Equal amounts of total lysates were resolved in SDS-PAGE and analyzed by immunoblot. Membranes were incubated with different primary and secondary antibodies and immunoreactive bands were detected by ECL.

### Calpain I *in vitro* proteolysis and prediction of calpain putative cleavage sites

Cell extracts were digested *in vitro* with purified calpain I or calpain II as reported.^26^ When indicated, 20 *μ*M CiIII or 0.5 *μ*M Tat-peptides were added to lysates immediately before protease addition. Calpain activation was established by immunoblot analysis through the detection of full length brain spectrin (FL) and its BDPs. Analysis of the potential calpain cleavage sites in rat Kidins220 last C-terminal 1000 amino acids was performed using the online services provided by Calpain Modulatory Proteolysis Database (CaMPDB; http://www.calpain.org) and GPS-Calpain Cleavage Detector (GPS-CCD; http://ccd.biocuckoo.org). Predictions from five different algorithms (Linear or Radial Basis Function Kernel, PSSM, MKL and GPS 2.0) were run. The results were compared for the identification of common putative cleavage sites present in Kidins220 C-terminus.

### Edman N-terminal sequencing of calpain cleavage sites

Total lysates from HEK293T cells expressing Kidins220-GFP-Ct fusion protein were digested with 80 U/ml of purified calpain I, as above. The C-terminal fragments generated after *in vitro* proteolysis were immunoprecipitated using a rabbit polyclonal anti-GFP antibody, resolved on SDS-PAGE, transferred to PVDF membranes and stained with Coomasie Blue R-250. The band of interest was cut, washed twice and kept at 4 °C until Edman degradation analysis. Sample duplicates were run in parallel and analyzed by immunoblot with the GFP antibody in order to ensure that electrophoretic mobility of purified bands matched that previously detected in HEK293T digestions. Edman N-terminal sequencing was performed by the Proteomic Service of the Centro de Investigaciones Biológicas (CSIC, Madrid, Spain). Six rounds of protein degradation followed by HLPC identification of excised amino acids revealed the sequence of interest.

### Assessment of neuronal viability in primary cultures

Measurement of neuronal survival by MTT reduction assay (Sigma Co.) was performed as previously described.^26, 31^ As we are using primary cultures containing cortical neurons and glial cells, we established the contribution of glia viability to total values by exposing sister cultures to 400 *μ*M NMDA, 10 *μ*M glycine for 24 h, conditions which induce nearly complete neuronal death and no glial damage. After subtracting this absorbance value, we obtained the viability of the neuronal subpopulation. For Calcein-AM/propidium iodide staining, neuronal cultures grown on Ibidi l-Slide 8-well chambers (IBIDI LLC Martinsried, Germany) were left untreated or stimulated with NMDA for 4 h. Then, culture media was replaced by serum-free Neurobasal medium (Invitrogen-Live Technologies) containing a mixture of 1 *μ*M Calcein-AM, a highly permeable non-fluorescent molecule transformed to fluorescent calcein dye in viable cells, and 0.5 mM propidium iodide which can only reach the nucleus of dead cells. Incubation proceeded for 15 min at 37 °C and neuronal viability was assessed by time-lapse microscopy using the Cell Observer Z1 (Carl Zeiss MicroImaging) at 37 °C and 5% CO_2_/95% air, AxioVision 4.8 imaging software and a Cascade 1 k camera. A minimum of five random fields and at least 500 cells were counted for each sample. Only cells with a clear neuronal morphology were considered for further analysis. The percentage of viable (green) or death (red) neurons was expressed relative to the total number (green+red) of cells examined per condition.

### Quantitative and statistical analysis

Immunoblot signals were quantified by densitometric analysis (NIH Image), normalized using NSE and expressed relative to values obtained in their respective controls. Results are shown as mean±S.E.M. of 3–7 independent experiments. Statistical significance was determined by Student's *t*-test. A *P*-value smaller than 0.05 was considered statistically significant: *P<0.05, **P<0.01, *** P<0.001.

## Abbreviations

ARMS: ankyrin repeat-rich membrane spanning
BBB: blood–brain barrier
BDNF: brain-derived neurotrophic factor
BDPs: breakdown products
CiIII: calpain-specific inhibitor III
CPP: cell-penetrating peptide
CREB: cAMP response element-binding protein
DIVs: days *in vitro*
dMCAO: distal occlusion of the middle cerebral artery
Eph: ephrin receptor
ERKs: extracellular signal-regulated kinases
Kidins220: Kinase D-interacting substrate of 220 kDa
NMDARs: *N*-methyl-d-aspartate type of glutamate receptors
PDZ-L: Postsynaptic density-95, Discs Large A and Zonula occludens-1 ligand
Trk: tropomyosin-related kinase
