# Supplementary figures and images for: Development of a neuroprotective peptide that preserves survival pathways by preventing Kidins220/ARMS calpain processing induced by excitotoxicity
Source: Cell Death Dis. 2015 Oct 22;6(10):e1939–. doi: 10.1038/cddis.2015.307 (PMC4632323; doi:10.1038/cddis.2015.307)

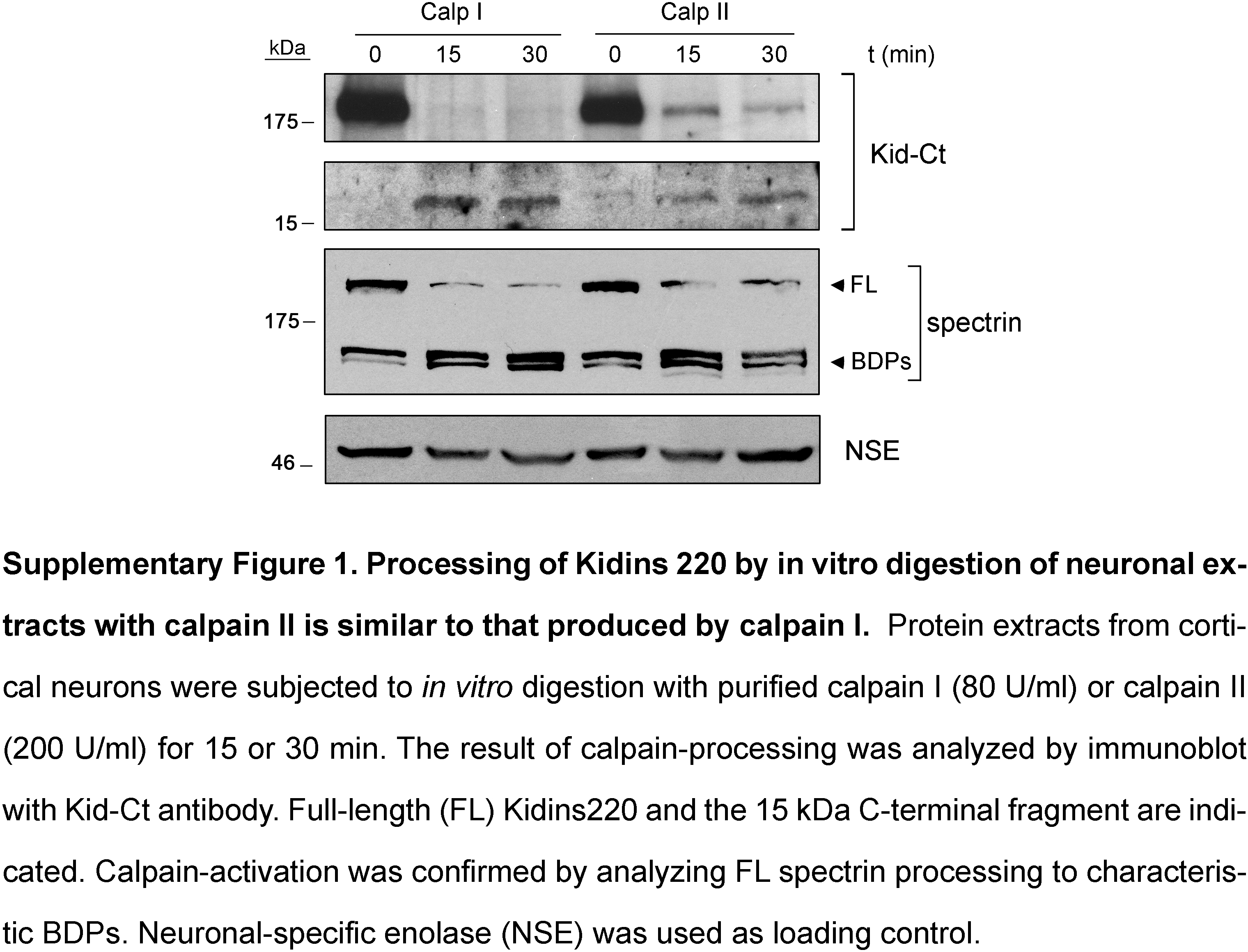

Supplement: Supplementary Figure 1 [file cddis2015307x1.tif]

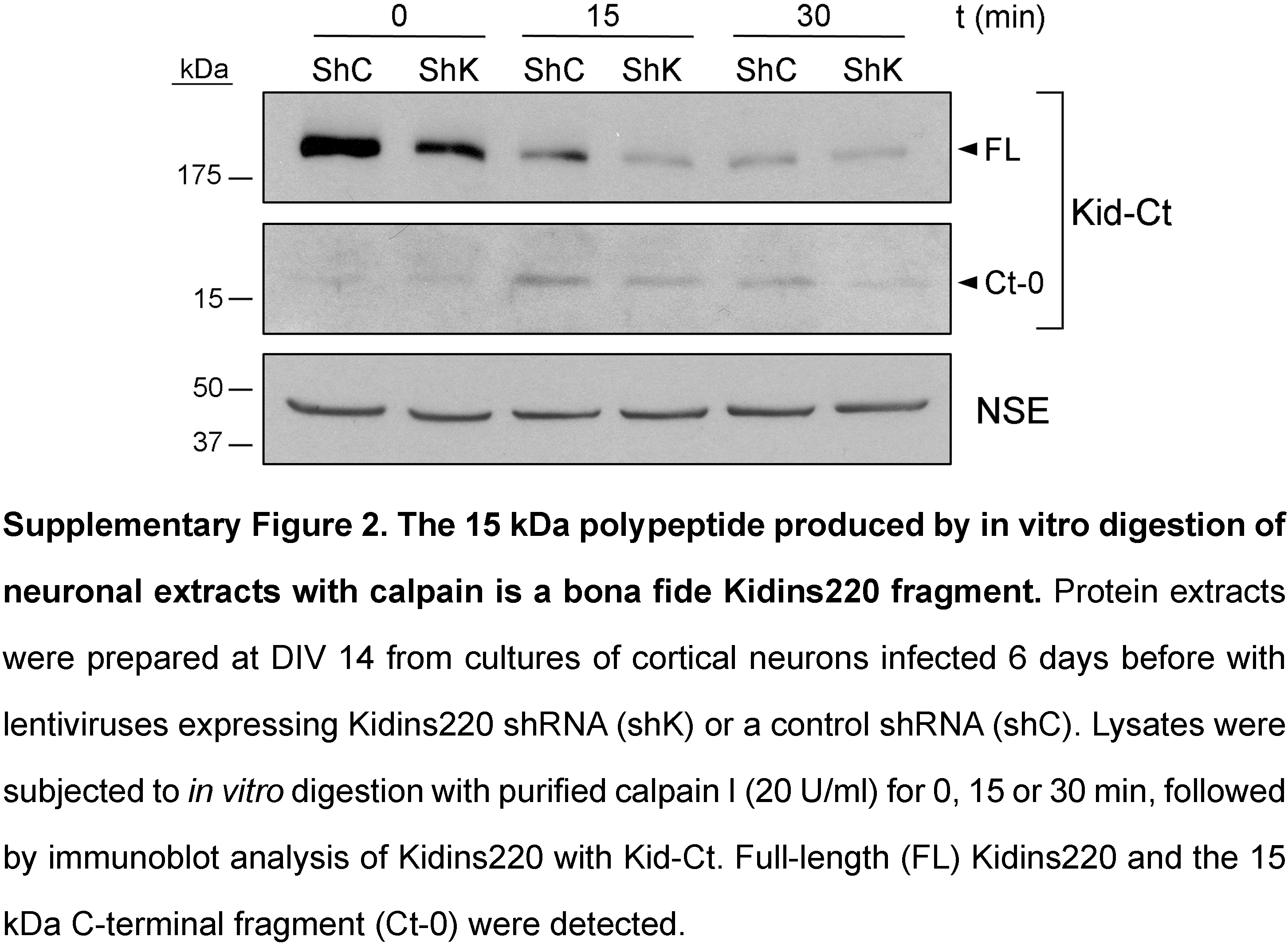

Supplement: Supplementary Figure 2 [file cddis2015307x2.tif]

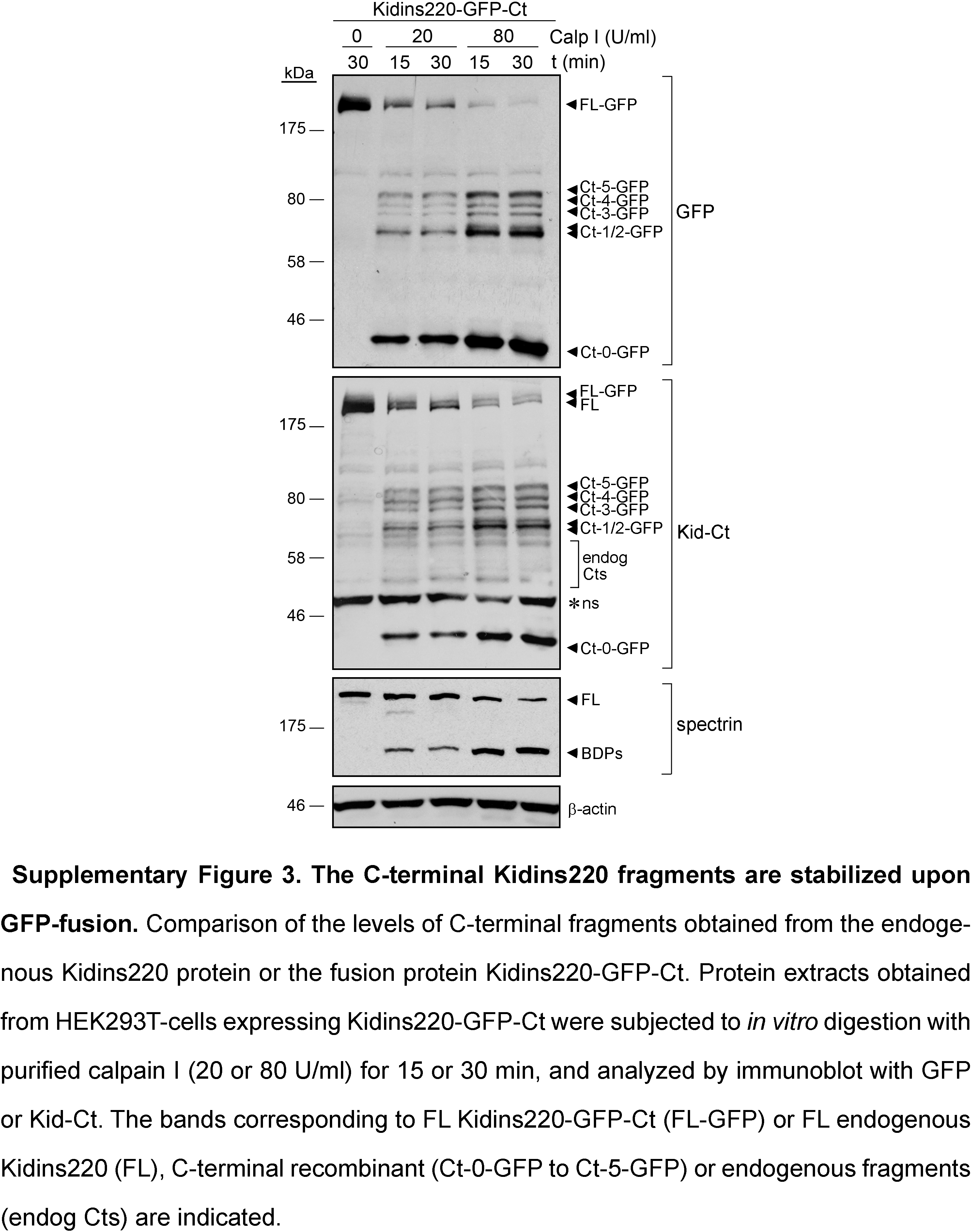

Supplement: Supplementary Figure 3 [file cddis2015307x3.tif]

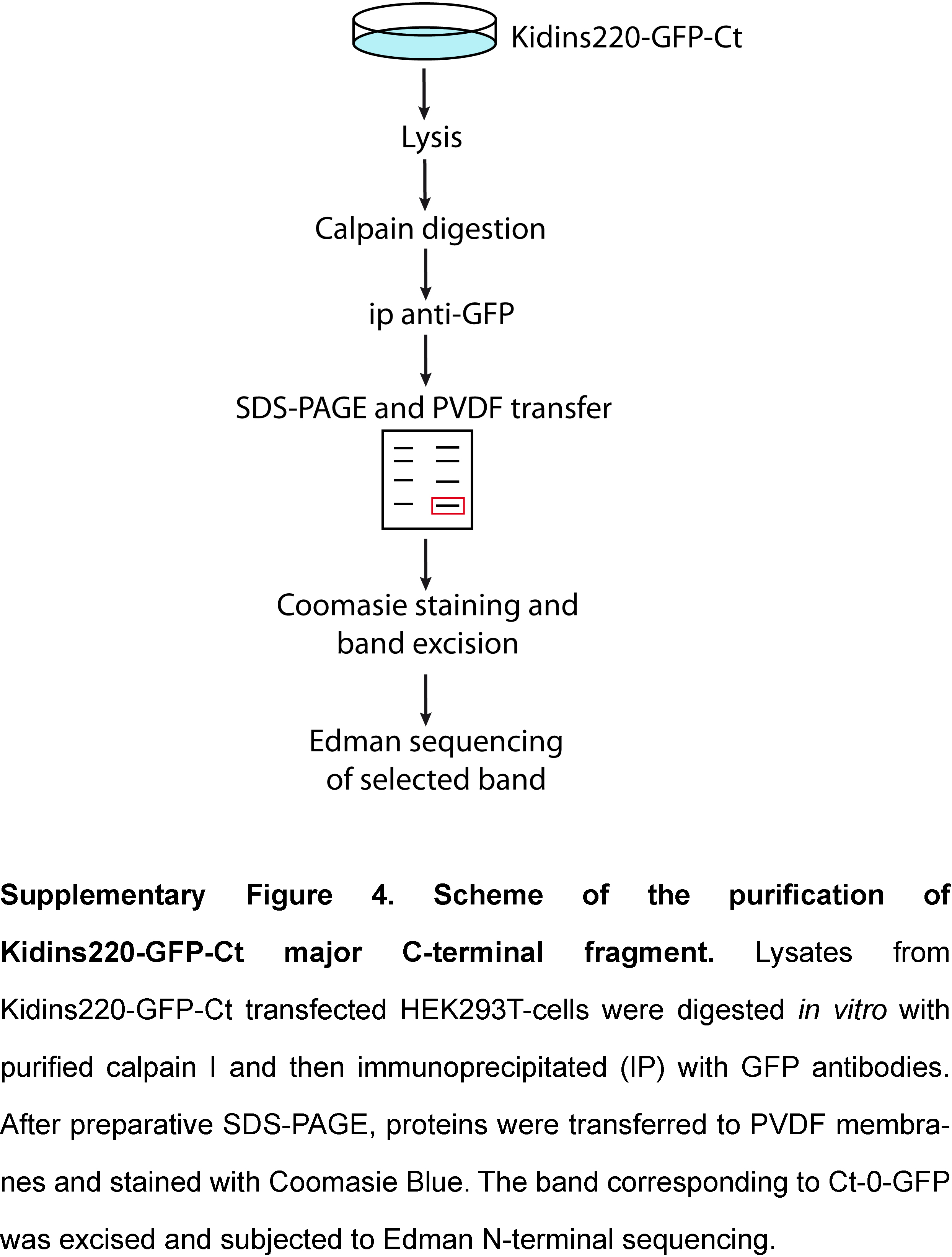

Supplement: Supplementary Figure 4 [file cddis2015307x4.tif]

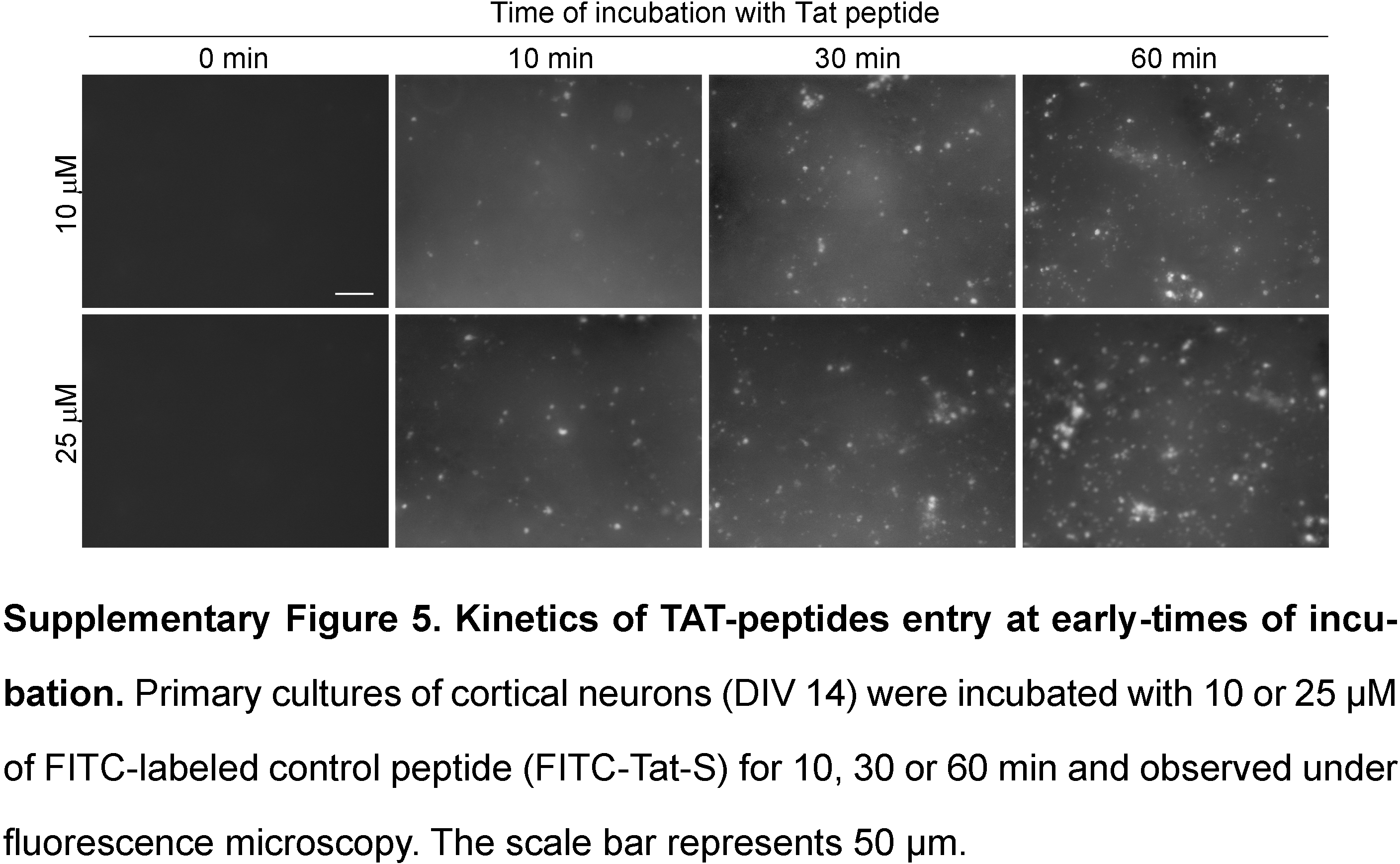

Supplement: Supplementary Figure 5 [file cddis2015307x5.tif]

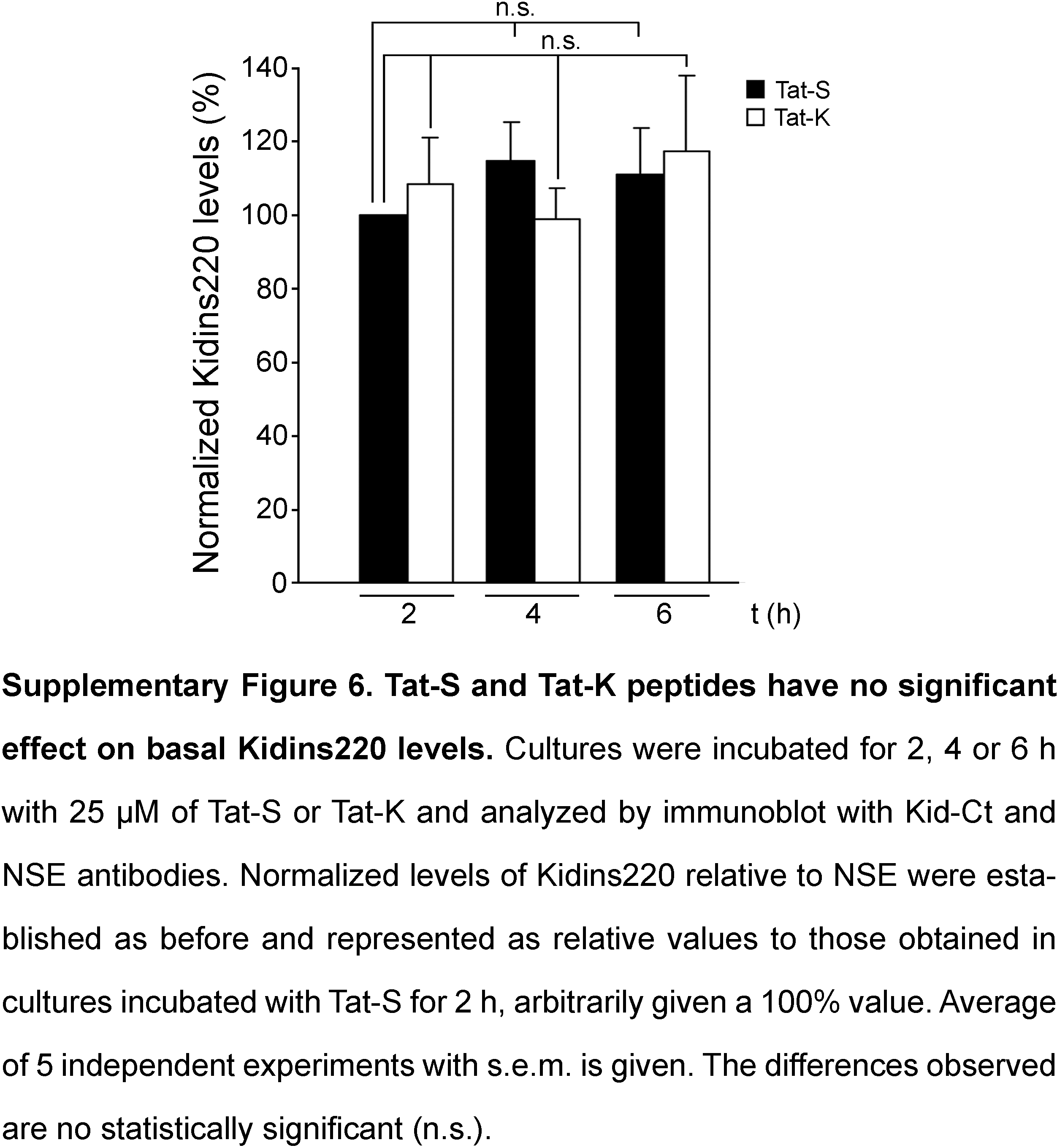

Supplement: Supplementary Figure 6 [file cddis2015307x6.tif]

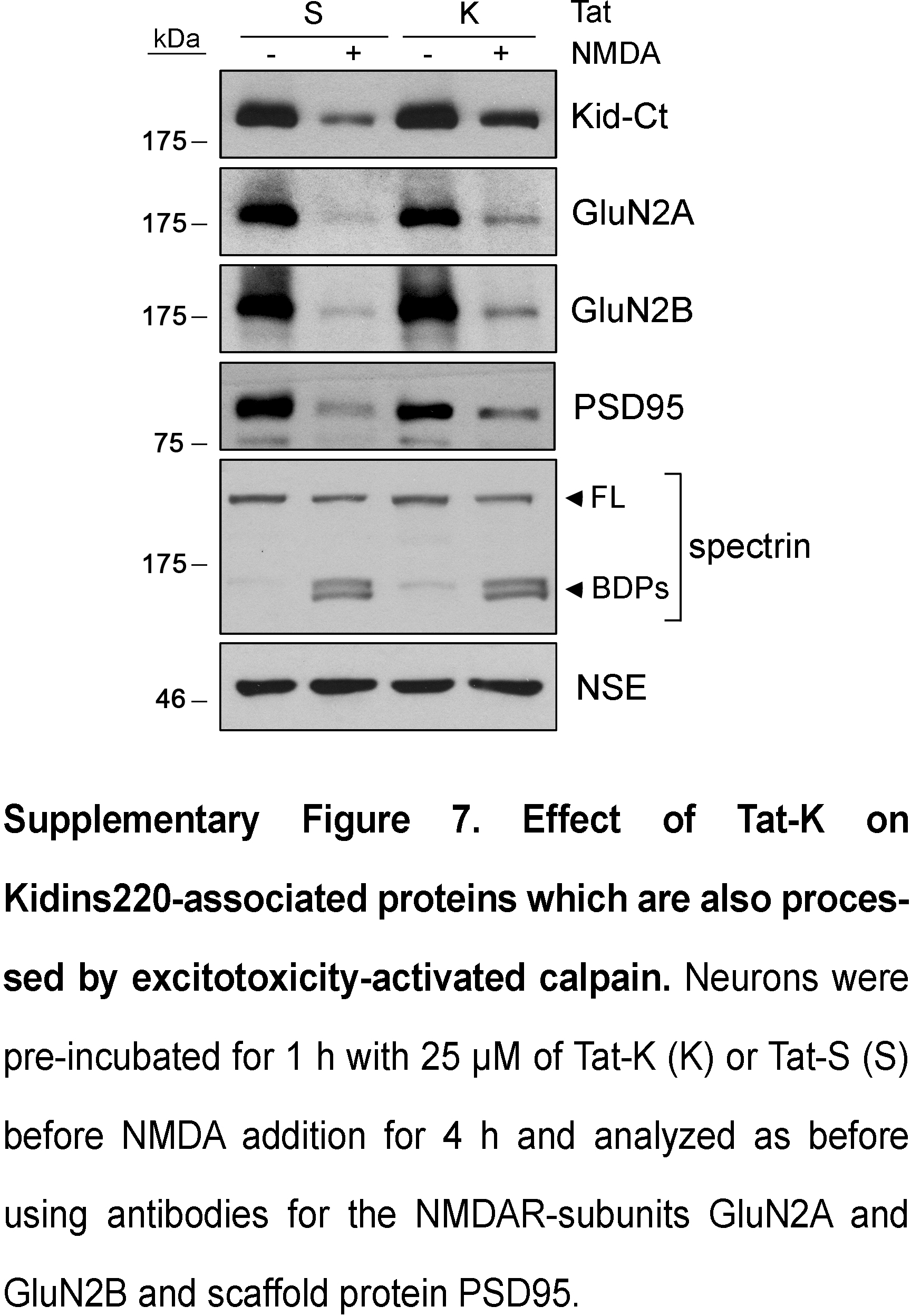

Supplement: Supplementary Figure 7 [file cddis2015307x7.tif]

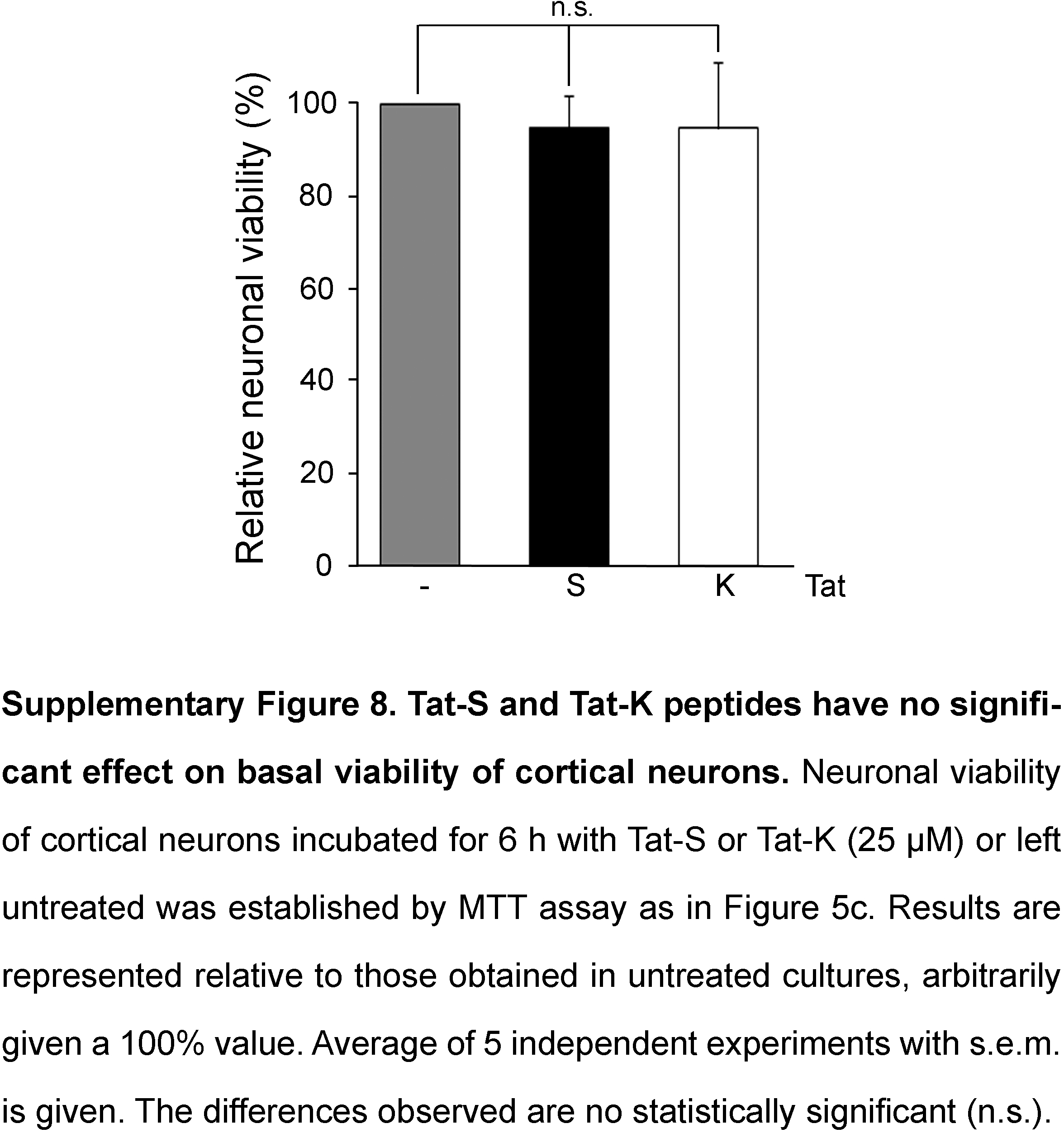

Supplement: Supplementary Figure 8 [file cddis2015307x8.tif]
